# Mesenchymal stromal cell therapy (REGENACIP^®^), a promising treatment option in chronic limb threatening ischemia – a narrative review

**DOI:** 10.1186/s13287-024-03957-0

**Published:** 2024-10-08

**Authors:** Sanjay Desai, Digvijay Sharma, Rajesh Srinivas, Venugopal Balaji, Vijay Thakore, Varinder Singh Bedi, Ravul Jindal, Amarnath Sugumaran, Senthilnathan Mohanasundaram, Jaideep Gogtay, Pawan Kumar Gupta, Aniruddha Bhuiyan, Gnaneswar Atturu

**Affiliations:** 1 Senior Consultant Vascular and Endovascular surgeon, Ramaiah Memorial Hospital, Bengaluru, India; 2 Head of Department, Vascular Interventions and Surgery, Fortis Escorts Heart Institute, New Delhi, India; 3 Vascular Surgeon, NH-Mazumdar-Shaw Medical Center, Bengaluru, India; 4 Senior Vascular Surgeon, Apollo Hospital, Chennai, India; 5 Senior Vascular Surgeon, Aadicura Super Speciality Hospitals, Vadodara, India; 6 Chairman & Senior Consultant, Institute of Vascular & Endovascular Sciences, Sir Ganga Ram Hospital, New Delhi, India; 7 Director of Vascular & Endovascular Surgery, Fortis Hospital, Mohali, India; 8 Medical Affairs, Cipla Ltd, Mumbai, India; 9 Stempeutics Research Pvt. Ltd, Bengaluru, India; 10 Consultant Vascular & Endovascular Surgery, Vascular Care n Cure, Mumbai, India; 11 Head & Senior Consultant, Department of Vascular & Endovascular Surgery, Renova Hospitals, Hyderabad, India

**Keywords:** Critical limb ischemia, Stem cell therapy, Angiogenesis, Buerger’s disease, Peripheral arterial disease

## Abstract

Chronic Limb Threatening Ischemia (CLTI) is a challenging clinical problem associated with high morbidity and mortality. Endovascular interventions have been the cornerstone of treatment whenever possible. It is estimated that CLTI represents < 10% of all Peripheral Artery Disease patients, yet 50% of the patients end up either with a major amputation of the lower limbs or die of cardiovascular causes within one year period, especially in those with unsuccessful revascularization or “no-option” CLTI. Cell-based therapeutics, especially bone marrow-derived mesenchymal stromal cells have emerged as a potential, promising, and novel alternate therapeutic modality in the management of CLTI, bolstered with positive results in numerous research, including randomized and nonrandomized trials. REGENACIP^®^ is one such BM-MSC therapy approved by Central Drugs Standard Control Organization in India for the management of “no-option” Atherosclerotic Peripheral Arterial disease / Buerger’s disease patients with established critical limb ischemia in Rutherford Grade III-5 or III-6, not eligible for or have failed traditional revascularization treatment, with rest pain and / or ulcers in the affected limb. The current review aims to deliberate upon the various aspects of CLTI and clinical benefits of REGENACIP^®^ therein.

## Introduction to chronic limb threatening ischemia

Peripheral artery disease (PAD) is described as the emergence of chronic arterial occlusive disease of the lower extremities due to atherosclerosis [1]. PAD is one of the main modes of expression of atherosclerosis, presenting with stenosis or occlusion occurring anywhere from the aortoiliac segment to the pedal arteries. Chronic limb-threatening ischemia (CLTI), also referred to as Critical limb ischemia (CLI) is the severe subset and end stage of PAD and is defined by severe pain at rest (lasting > 2 weeks), and/or non-healing ischemic skin lesions and/or gangrene of the extremity due to inadequate blood supply. Non-Atherosclerotic causes of CLTI include Thromboangitis Obliterans (TAO), also known as Buerger’s Disease (BD), which is a recurring progressive inflammation and thrombosis of small and medium arteries and veins of the hands and feet [2]. It is estimated that CLTI represents < 10% of all PAD patients, yet 20% of the patients suffering from CLTI will end up either with a major amputation of the lower limbs or die of cardiovascular causes within one year period [3]. Comorbidities like diabetes, obesity, chronic kidney disease particularly end stage renal disease, hypertension, dyslipidemia, cardiovascular diseases particularly prior MI, stroke or heart failure, lifestyle disorders, smoking habit, family history of PAD, increasing age are some of the significant risk factors for PAD which may progress to advanced atherosclerosis and development of CLTI [3, 4]. The risk of myocardial infarction and stroke is 30–50% over a year; major amputation (at or above the ankle) is less than 5% over 5 years in patients with claudication while it is 30–50% in the first year in patients with CLI who did not undergo revascularization [5, 6].

### Epidemiology of peripheral artery disease and CLTI

The occurrence of PAD is progressively on the rise, and it is now gaining recognition as a leading factor contributing to cardiovascular morbidity and mortality. During the period 2000 to 2010, studies reported 200 million people to be affected with PAD worldwide [7]. These numbers increased to 236 million adults in 2015, with a global prevalence of 5.6%. The low- and middle-income countries contributed to 73% of the PAD affected population. Thus, there was a 45% increase in the global PAD prevalence (~ 18% in higher income countries and ~ 58% in low and middle-income countries). In the US, 8.5 million adults are reported to live with PAD, reflecting a prevalence of 7% [8]. Studies in Europe have suggested a higher prevalence of PAD at 7.99% in the European region [9]. PAD prevalence was higher among women and incidence and prevalence were found to be strongly affected by age. According to the 2019, Global Burden of Disease data, PAD was documented to impact 332.32 per 100,000 males and 621.11 per 100,000 females within the age of 40–44 and notably increased to 17,195.57 in males and 24,965.3 in females aged 95 and above [9]. Generally, PAD prevalence increases steadily above the age 65 years. CLTI constitutes nearly 1% of the adult population and its prevalence increases to 10% among elderly population with PAD, with an annual estimated incidence of 220–3500 new cases per million population. It has been reported that 5–10% of patients with asymptomatic PAD or intermittent claudication will progress to CLTI over five years [10]. CLTI prevalence in the US population > 40 years is estimated to be 1.28%, which is approximately 2 million total CLTI patients with an annual incidence range from 0.26 to 0.48%. Amputation rates may vary among patients typically exceeding 15–20% in the first year and reaching values of up to 67.3% at four-year follow-up in patients with more advanced disease. This ultimately affects not only limb loss but also in-hospital and long-term mortality, which over five years is usually above 50% [10].

Research on the epidemiology of CLTI is limited with little or no population-based data on the number of people suffering from PAD and/or CLTI in India. In the Strong Heart Study (Kerala, India) with participants aged 45–74 years, PAD was seen in 5.3% [11]. An independent, small population-based observational study in South India reported a prevalence of PAD as 7.6%, with women exhibiting a prevalence of 11.8% and men 5.1% [12]. An independent international organization report estimated that the prevalence of peripheral artery disease in India ranges from 41 to 54 million, with 4.2 to 6.2 million people affected by CLTI. Diabetes is one of the major risk factors for the development of PAD, affecting 60–80% of patients with CLTI [13, 14]. Moreover, with diabetes striking a decade earlier among the Indian population, the onset of PAD among younger Indians is high and remains mostly underdiagnosed [14]. As PAD progresses to CLTI, the risks of limb loss (40% experience amputation) and mortality increase (20%) [15].

### Epidemiology of Buerger’s Disease and CLTI

Buerger’s Disease is a rare condition with an unclear etiology that occurs worldwide. Its estimated global prevalence ranges from 5 to 12 per 100,000 people per year, although this can vary significantly by region, with incidences reported from as low as 0.25 to as high as 693 per 100,000 people per year [16]. Its prevalence among patients with peripheral vascular disease is most reported among the Asian population of Middle and Far Eastern nationalities like Korea and Japan (16 to 66%), India (45 to 63%), Israel (80%) as compared to Europe (0.5 to 5.6%) Latin America (11.2%) [2] and North America (12.6–20 per 100,000 individuals) [17]. It is predominantly reported among young male smokers however, its incidence among female smokers is increasing (11–23%) [18]. Pediatric and geriatric population are not generally afflicted with BD [17]. Most patients with BD are aged 22–45y with men to women ratio 3:1 [17, 19]. The risk of amputation in patients with BD is largely associated with the continuation of smoking. A national study in Japan showed 2.73 times higher risk of amputation among BD patients who continued smoking [20]. The amputation risk remains high among continued smokers with 25% patients undergoing amputation at the end of 5y and 45% at the end of 10y period [18–20]. 

### Pathophysiology of CLTI

**Fig. 1:**
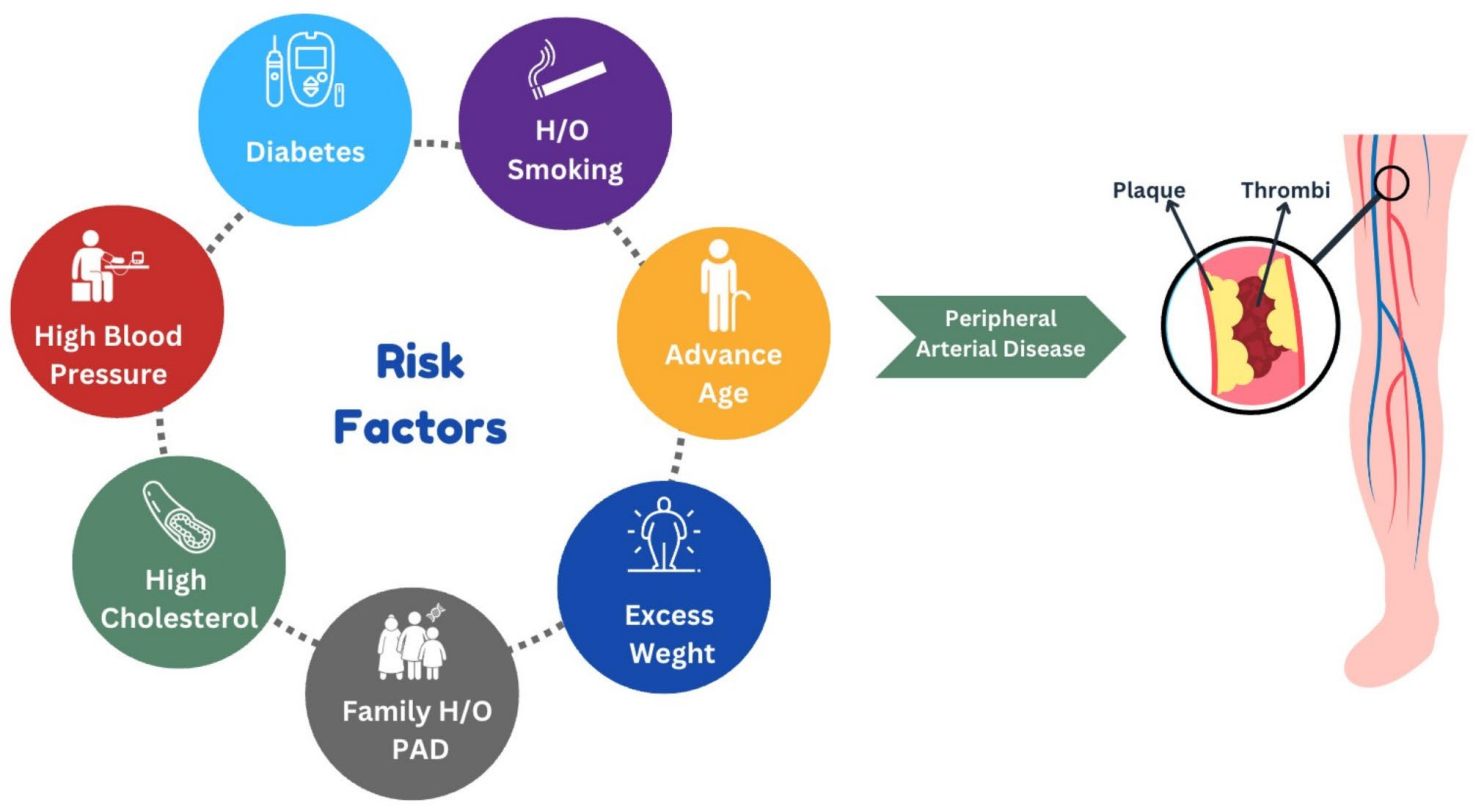
Risk factors governing Peripheral artery disease [21]

CLTI is the more severe form of PAD associated with a high risk of limb loss. PAD and BD are the two main causes of CLTI. PAD is a result of atherothrombotic narrowing and occlusion of the lower limb arteries. PAD usually involves atherosclerotic blockade in the abdominal aorta, iliac, femoral, tibial and popliteal arteries. In the initial phases of PAD, there is a gradual accumulation of plaque on the inner walls of arteries. This promotes complex changes in the lower limb circulatory vasculature by dilating the collateral blood vessels or genesis of newer blood vessels to provide alternative blood flow around the affected arterial segment. As the blockage becomes progressively more severe, the blood flow cannot meet the resting metabolic demands of the lower extremity leading to ischemic rest pain, burning pain in the soles of the feet, and ischemic ulcers. Several risk factors like smoking, increasing age, diabetes mellitus, dyslipidemia, and CAD are associated with the development of PAD, as shown in Fig. 1 [21]. Multiple occlusive lesions in the limb arteries accompanied by functional and structural changes in the microcirculation result in inadequate tissue perfusion, skin ulcers, and necrosis. In CLTI, the arterial occlusions are higher in number and more distal than found in claudication and alter the blood flow and oxygen delivery to distal tissue leading to serious hemodynamic compromise. Tibial vessels are the most affected in CLTI [22].

### Management of CLTI

**Fig. 2:**
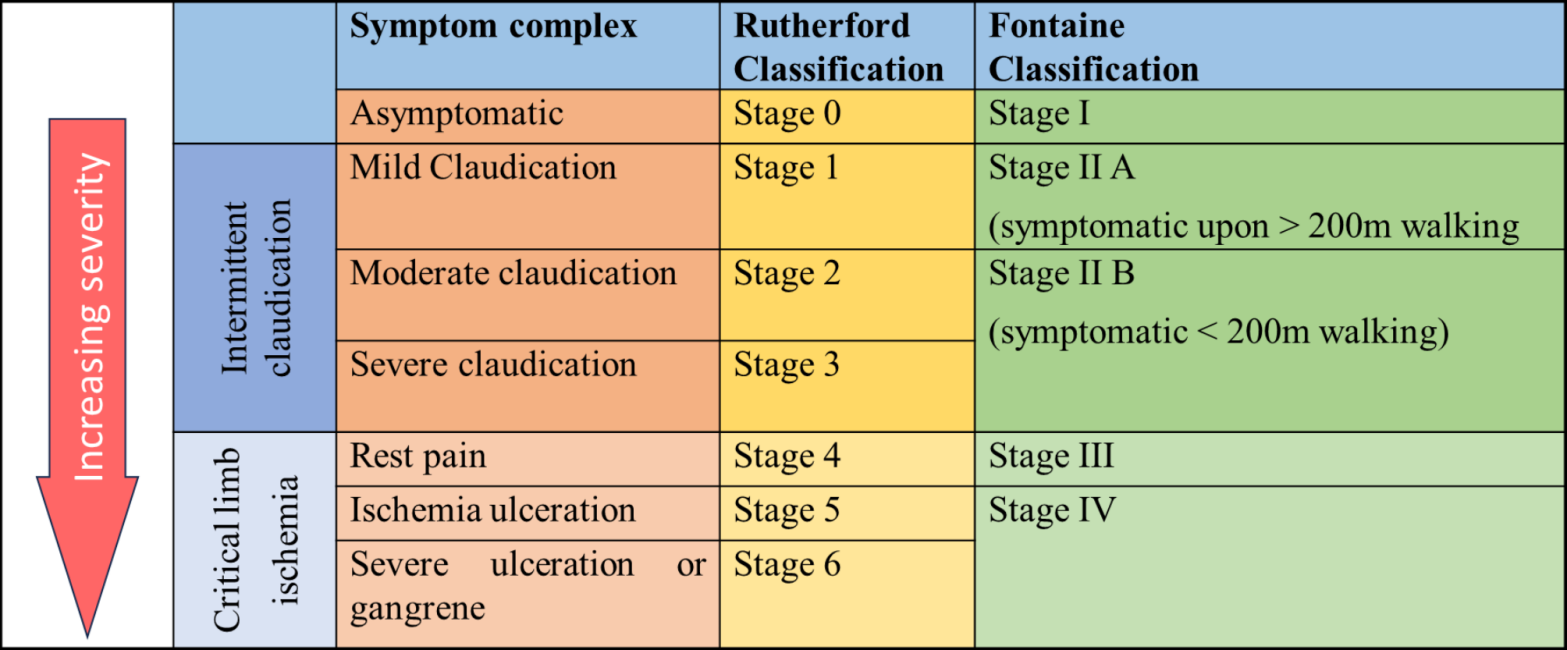
Classification system of PAD [24]

PAD is mostly diagnosed on clinical grounds in patients with lower limb symptoms of intermittent claudication, rest pain, ulcers, numbness, or tissue loss in the distal leg or foot. Clinical examination in these patients includes examining the distal peripheral pulses, ankle pressure (AP) and measurement of the ankle brachial index (ABI). Objective walk test and medical imaging support the differential diagnosis in patients with clear evidence of CLTI [23]. The different classification systems of PAD have been summarized in Fig. 2 [24]. The Fontaine classification system assigns severity based entirely on clinical symptoms without other diagnostic tests. Rutherford classification assigns severity based on performance on the 5-minute treadmill test at 2 mph on a 12% incline. 

### Current treatment modalities in CLTI

Interventions ought to be considered where comprehensive guidelines directed risk factor control and medical therapy (GDMT) has failed, and the severity has progressed to CLTI. Intervention may be endovascular (angioplasty or stenting) or open surgical bypass. Recent advances in the Endovascular techniques have expanded the indications for minimally invasive interventions in CTLI. Longer segments and multi-level disease CTLI are now being treated endovascularly. Surgical bypass is generally reserved for patients with debilitating claudication and in patients wherein angioplasty is unlikely to have better outcome [25].

### Limitation of current treatment modalities

Surgical revascularization aims to minimize the need for amputation by enhancing blood flow to the foot, thereby facilitating wound healing. However, high-risk patients carry significant morbidity and mortality risk. In addition, intimal hyperplasia at the site of bypass is a major threat to graft patency with studies quoting one year and two-year patency off [26]. Endovascular interventions enjoy the benefit of being less invasive, reducing morbidity and mortality. However, restenosis or occlusion at the angioplasty site is inevitable. Short and long-term results from balloon angioplasty are suboptimal with a 1-year primary patency rate at 63% [27]. Restenosis was reported to occur in up to > 60% of CLI patients undergoing angioplasty of complex tibial arterial obstructions [26]. Large vessel interventions have better patency with studies showing 5 years patency rate of, 79% after iliac angioplasty and 55% after femoral angioplasty [25]. There is no standard for revascularization strategies or the extent of revascularization while treating CLTI patients. Many high-risk patients with multivessel disease, multimorbid and risk of CAD may not be suitable for revascularization interventions. Moreover, revascularization alone may be inadequate, demonstrating a 6–10% amputation rate even in patients with patent bypass grafts [26, 27].

### Stem cell-based therapy as a potential alternative to current therapy

Stem cell- based approaches have now emerged as novel therapeutic alternatives for different diseases where pharmacological therapies and surgical interventions have limited effect, like CLTI, due to their angiogenic role, and their regenerative and immunomodulatory effects on tissue lesion [10]. Two categories of stem cells exist: adult stem cells, which are sourced from various body tissues like adipose tissue, skin, dental pulp, and primarily bone marrow and embryonic stem cells that are produced from an early embryo. Unlike embryonic stem cells, which can become any cell in the body (called pluripotent), adult stem cells are usually restricted to become any type of cell in the tissue or organ that they reside (called multipotent) [28]. Bone marrow derived stem cells are considered as the gold standard and currently the most widely employed, regenerative, adult cell based therapeutic strategy, thus becoming exceptionally valuable in guiding the management of acute and chronic limb ischemia [28].

### Mesenchymal stromal cells

**Fig. 3:**
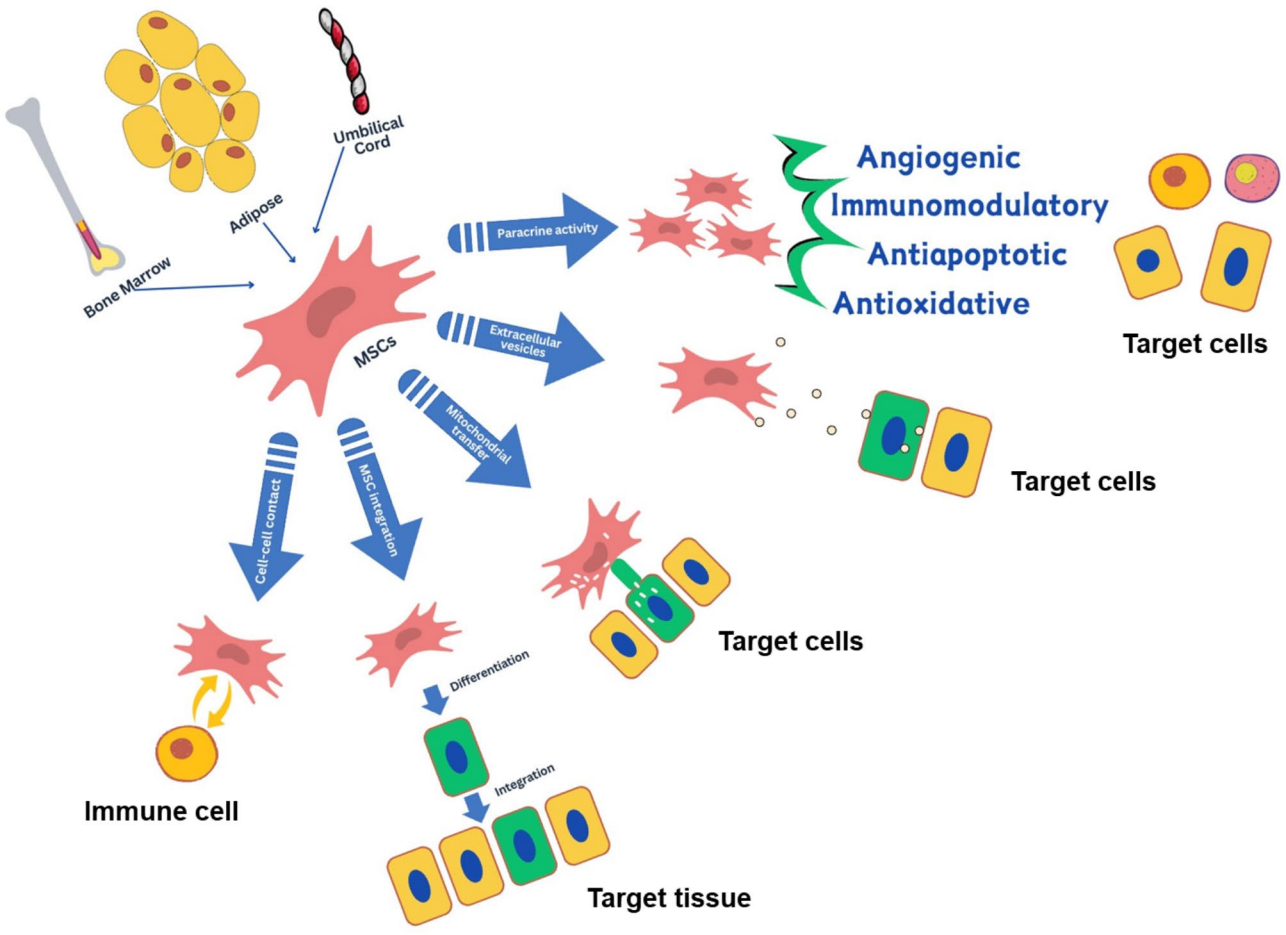
Molecular mechanism of MSC

Mesenchymal stromal cells (MSC) are multipotent stromal cells that can regenerate and differentiate into various cell types. They secrete a high level of paracrine factors, demonstrate enhanced proliferation and differentiation potential, showcase anti-inflammatory properties, possess increased resistance to inflammation, along with no teratogenic/carcinogenic potential. They have multiple mechanisms of action, predominantly facilitated by paracrine functions that secrete an array of soluble factors to elicit immunomodulatory, angiogenic, antiapoptotic, and antioxidative effects [29–33]. The molecular mechanistic aspects of MSC has been illustrated in Fig. 3.

The clinical efficacy of MSC can differ significantly based on factors such as the tissue source, donor characteristics, as well as preparation and administration protocols. Factors specific to the donor, including age, sex, body mass index, and underlying health conditions, can affect the phenotype, morphology, differentiation potential, and function of MSC [34]. Additionally, variations in MSC preparation contribute to heterogeneity due to the composition of cell culture media, the inclusion or exclusion of growth factors, the use of various serum supplements, and differing culturing techniques [34]. The significant variability observed in MSC secretomes across these parameters highlights the need for standardizing and optimizing protocols [35].

### Safety, regulatory and ethical consideration for stem cell therapy

MSCs based therapy are faced with some safety concerns, particularly regarding long-term implications, focusing on the unintended differentiation of transplanted MSCs, their ability to inhibit anti-tumor immune responses, and their potential to induce angiogenesis that may facilitate tumor progression and metastasis. MSCs possess the capacity to differentiate into unintended tissues, such as bone and cartilage [36]. MSCs easily gather together, forming the core of clots and leading to vascular disorders [37]. Wang et al. performed a meta-analysis to identify all treatment-related adverse events concerning MSC administration and explore the safety of MSC in clinical utilization which included 62 high-quality studies, and data from 3546 patients for 15 years. No serious adverse events such as death and infections were reported in any of the studies. The analysis of the pooled data showed transient fever, administration site adverse events, constipation, fatigue and sleeplessness as common adverse events associated with MSC treatment. Thus, the study confirmed the safety of MSC in different populations compared to placebo [37]. 

The guidelines of regenerative technologies is regulated in most developed countries. The Food and Drug Administration (FDA), the Office of Therapeutic Productswithin the FDA Center for Biologics Evaluation and Research (CBER) currently regulate cell-based therapies in the United States of America [38]. India is one of the select few nations that have established comprehensive and formalized guidelines pertaining to the ethical and procedural framework governing research activities involving stem-cell based products. In this regard, The National Guidelines for Stem Cell Research was jointly drafted by Indian council of medical research and department of Biotechnology, India and released in 2017 with the aim of facilitating safe, ethical, and regulated translational and clinical stem cell research in India [39, 40]. As per these guidelines, stem cell research pertaining to establishment of new embryonic stem cells or induced pluripotent stem cell lines is permissible while research involving human germline gene therapy, reproductive cloning is prohibited. All translational research with clinical studies must obtain mandatory approval from the ethics committee, regulatory body and establish a data and safety monitoring board to ensure safety of the participants. All the Adverse events emerging in these studies must be reported to the regulatory authorities. The commercial use of stem cell therapy must be as per the intended use as approved by regulatory authorities with proven clinical benefits and after obtaining proper consent [38–40]. Owing to the lack of regulatory enforcement in many parts of the world, there are ethical concerns with the preparation and moral use of stem cell-based therapy. The ethical concerns majorly arise from the harm of the unproven stem cell interventions, side effects, improper storage of donated stem cells, the discussion with ownership. This necessitates the need for stronger regulation governing the safe, ethical and effective use of MSC [41]. 

### REGENACIP - bone marrow mesenchymal stromal cells

REGENACIP^®^ is an innovative formulation of human mesenchymal stromal cells sourced from the bone marrow of unrelated healthy adult donors, developed in India.

### Regulatory approval status of REGENACIP

REGENACIP^®^ is the first stem cell-based biologics to be approved by Central Drugs Standard Control Organization [CDSCO] in patients with Atherosclerotic Peripheral Artery Disease patients or Buerger’s disease with established critical limb ischemia in Rutherford III-5 or III-6, not eligible for or have failed traditional revascularization treatment, with rest pain and/or ulcers in the affected limb [42].

### Preparation of REGENACIP

The preparation of REGENACIP follows a well-documented and patented technology [43, 44]. Briefly, healthy volunteers for bone marrow donation are screened and bone marrow derived MSC are isolated and cultured in vitro with the help of the unique patented technology. A two-tier cell banking system is adopted wherein a donor master cell bank is created from individual donors and subsequently, the working cell bank is created by pooling MSC from three healthy donors. Each of the cell banks is cryopreserved and maintained at -185 °C to -196 °C. REGENACIP^®^ is manufacturing under aseptic conditions and strict Good Manufacturing Practice guidelines and qualified with stringent quality checks against the predefined specifications like identity, purity, impurity, potency, sterility, safety, and genetic stability, before releasing [39]. This unique patented manufacturing technology produces a wider repository of growth factors with the shelf life of the cryopreserved product being 18 months.

In REGENACIP^®^ formulation, the cells are cryopreserved (150 million (Mn) cells or 200 Mn cells) in 15 mL of multiple electrolytes solution containing 5% human serum albumin (HSA) and 10% Dimethyl sulfoxide (DMSO) in a Cryocyte bag and maintained at -185 °C to -196 °C. For administration, the cells are thawed by immersing the cryocyte bag in sterile distilled water maintained at 37 °C.

### Intramuscular administration of REGENACIP

REGENACIP^®^ administration procedures need to be done under intravenous sedation with cardio-respiratory monitoring. Premedication treatment generally involves administration of appropriate intravenous medication (100 mg Hydrocortisone and 45.5 mg of Pheniramine maleate) within 20–40 min prior to intramuscular REGENACIP^®^ injection [41].

**Fig. 4:**
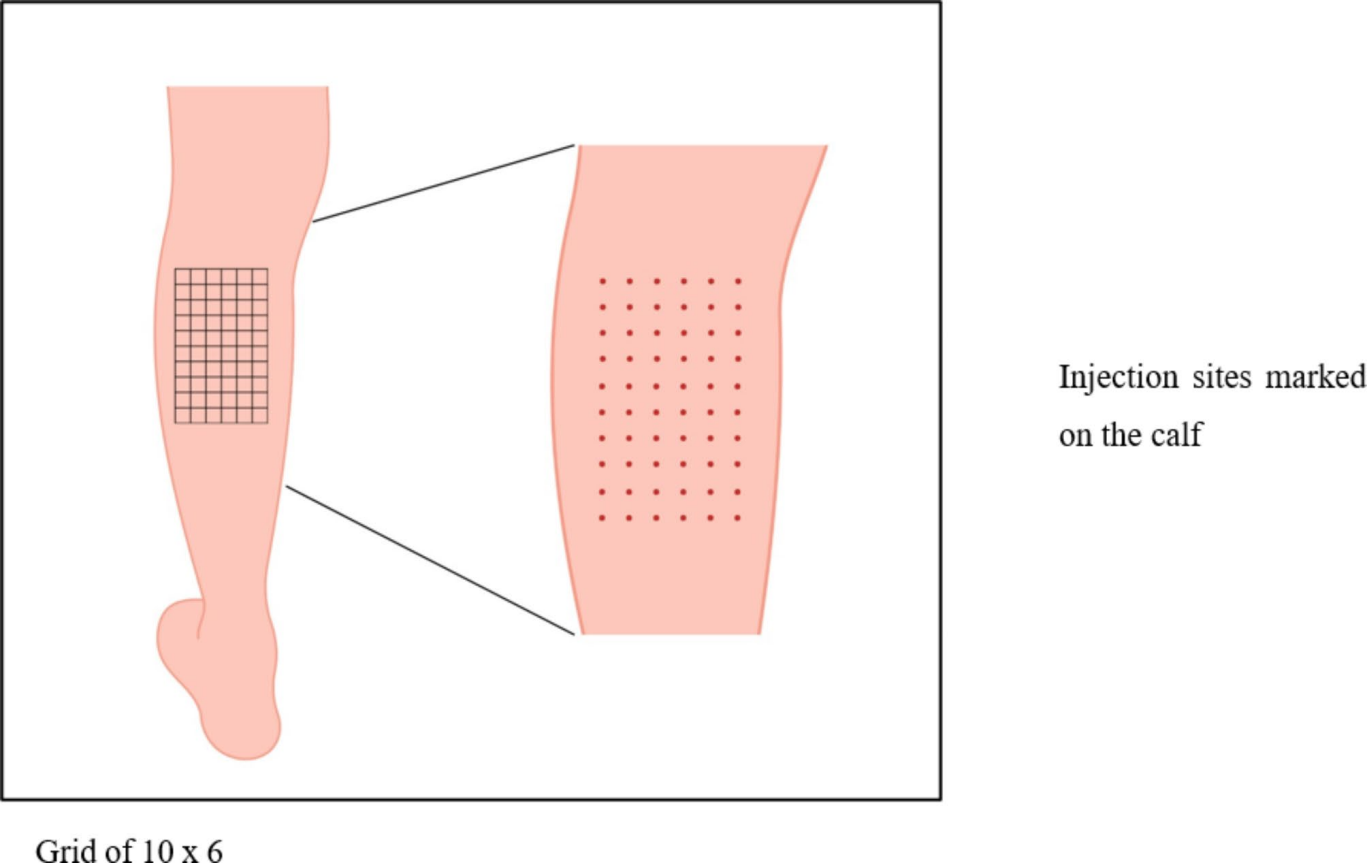
Representation of injection grid 10 × 6 cm for 60 injection sites

The dose of REGENACIP^®^ to be administered is calculated based on the body weight as 2 Mn cells/Kg body weight. Guide for dosage calculation based on some prespecified body weight has been enlisted in Table 1. The calculated volume of cell suspension is administered using 40–60 intramuscular injections in the gastrocnemius muscle of the ischemic lower limb (40–60 sites, distributed in an area of 10 × 6 cm, and 0.5-1.0 mL of REGENACIP^®^ per site). as shown in Fig. 4. While treating an ulcer, multiple peri-ulcer injections are (4–6 sites; 0.3–0.5 mL of REGENACIP^®^ per site) administered around the ulcer [44].

**Table 1:** Guide for dosage calculation based on some prespecified body weight

Patient weight in Kg	No of MSC’s required in millions.	Total volume to be administered from cell suspension.	Volume to be given by IM injection.	IM − 0.5 ml injections (no.)	IM − 1 ml injections (no.)	Total IM injections (no.)	Volume of local injection
50	100	30	28	44	6	50	2
60	120	36	34	32	18	50	2
70	140	42	40	20	30	50	2
80	160	48	46	8	42	50	2

Abbreviations: MSC, mesenchymal stem cells; IM, intramuscular

### Clinical pharmacology of REGENACIP

REGENACIP^®^, being MSC, primarily stimulate angiogenesis by secreting angiogenic cytokines and also by differentiating into endothelial cells. The mechanism of action of is likely due to a combined effect of anti-inflammation and pro-angiogenic activity governed by paracrine function or by directly producing the factors (VEGF, angiopoietin, IL-6, IL-8, and PGE2 amongst others) at the site of inflammation and ulcer location. REGENACIP^®^ may also stimulate the migration of host endothelial cells to the ischemic tissues which in turn lead to neo-angiogenesis by MSC which integrate to form new blood vessels [42, 43]. 

### Clinical studies

**Fig. 5:**
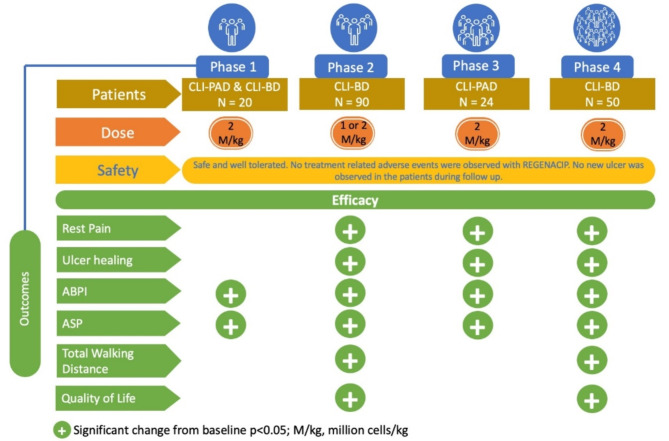
Overview of the clinical studies with REGENACIP^®^

REGENACIP has evidence at all phases of clinical trials and the overview of all these clinical studies has been represented in Fig. 5.

#### Phase I/II studies

Gupta et al. conducted a multi-center, placebo-controlled Phase I/II study (NCT00883870) to assess the safety and efficacy of intramuscular administration of REGENACIP^®^ in CLTI patients (*n* = 20) with infra-inguinal arterial occlusive disease and who were not suitable for or had failed revascularization treatment. All the patients had PAD with underlying etiology as either atherosclerosis or TAO. REGENACIP^®^ at a dose of 2 Mn cells/kg was administered intramuscularly at the gastrocnemius muscle of the ischemic limb while the placebo arm received multiple electrolyte solution. The administration of REGENACIP^®^ was well tolerated and no infection, bleeding, or procedural complications. There was no statistically significant difference in the overall incidence of adverse events between the two treatment arms. Clinical efficacy was evaluated using ankle pressure and ankle brachial pressure index (ABPI) parameters. Significant improvement in the mean ankle pressure from baseline (18.96 Vs. 3.92 mm Hg, *P* = 0.047) and mean change in ABPI (0.22 Vs. 0.00, *P* = 0.0018) was reported between treated and placebo arms, at the end of 6 months [43].

#### Phase II studies

In another study by Gupta et al. a phase II, nonrandomized, dose-ranging study (NCT01484574) was conducted prospectively to assess the efficacy and safety of intramuscular REGENACIP^®^ injections in patients with CLTI due to Buerger’s disease who had not responded to or were not eligible for a revascularization procedure. The majority of the study patients were young (38–42 years) and ex-smokers, and all patients had at least one ulcer. The study enrolled 90 patients who were allocated into three groups: group A and B received REGENACIP^®^ doses of 1 Mn cells/kg body weight (*n* = 36) and 2 Mn cells/kg body weight (*n* = 36) respectively, and group C received standard of care (*n* = 18). Group B exhibited statistically significant efficacy outcomes with reduction in mean pain score (7.03 Vs. 6.66 units; *P* = 0.019), healing of ulcers (mean ulcer area 4.09 Vs. 1.78 cm^2;^*P* = 0.025) and improvement in ABPI (0.45 Vs. 0.66; *P* = 0.013), while group A showed numerical improvement but statistically insignificant changes, as compared to group C [44]. Based on the clinically beneficial results with REGENACIP^®^ therapy, Indian regulatory authorities granted the marketing approval for REGENACIP^®^ in the indication of CLTI due to Buerger’s disease.

#### Phase III study

Gupta et al. conducted a single arm, multicentric phase III study (CTRI/2018/06/014436) to assess the efficacy and safety of REGENACIP^®^ in patients with CLTI due to atherosclerotic PAD. The study included 24 participants diagnosed with unilateral lower extremity CLTI caused by PAD. Each participant received an injection of REGENACIP^®^ at a dosage of 2 Mn cells/kg body weight into the gastrocnemius muscle. Throughout the study duration of 12 months, there was gradual decrease in rest pain scores, with statistically significant reduction in mean rest pain scores from 8.0 at baseline to 1.1 at the end of study (*P* < 0.0001). Out of 28 ulcers which were noted at the baseline, 82% were completely healed within 12 months with REGENACIP^®^ therapy. No new ulcer was observed in any of the patients during the 12 months of study. The mean ulcer area also showed significant reduction from 2.06 cm^2^ at baseline to 0.46 cm^2^ (*P* < 0.0001) after 12 months. The mean ABPI showed significant improvement from 0.47 at baseline to 0.73 at 12 months. The assessment of improvement in mobility was done by measuring the total walking distance calculated with the formula:

Total Walking Distance = Total Walking time [Min] X 3.2 [Km/h] **/** 60 [Min].

Total walking time was assessed based on patients’ ability to walk on a treadmill at 2 mph (3.2 km/h) with inclination up to 12% grade, until they stopped because of claudication or till maximum walking time of 60 min. None of the patients underwent major amputation during the study. Based on the statistically significant clinical benefit, the India regulatory authorities granted the marketing approval to REGENACIP^®^ in CLTI patients with PAD [45]. The summary of efficacy parameters of phase III trial is summarized in Table 2; Fig. 5.

**Table 2:** Summary of statistically significant change in efficacy outcomes at 12 months with REGENACIP ^®^ therapy in phase III trial performed in CLTI patients due to PAD [45]. [The change from baseline in each parameter is statistically significant at *P* < 0.0001.]

Sr No.	Efficacy parameter	Baseline	1 month	3 months	6 months	12 months
**1**	Rest pain score	8.0 (1.57)	4.6 (2.17)	2.8 (1.87)	1.7 (2.22)	1.1 (1.80)
	Absolute Mean (SD) Change from baseline	-	3.3 (2.11)	5.2 (2.19)	6.3 (2.72)	6.9 (2.53)
	% improvement from Baseline	-	43.9	64.5	78.1	84.9
**2**	Ulcer size (cm^2^)	3.98 (2.524)	2.06 (2.104)	1.13 (1.509)	0.64 (2.126)	0.46 (2.124)
	Absolute Mean (SD) Change from baseline (cm^2^)	-	1.92 (1.933)	2.85 (2.301)	3.34 (3.223)	3.52 (3.211)
	% improvement from Baseline	-	48.4	63.2	79.7	88.6
**3**	Ankle systolic pressure (mmHg)	61 (22.1)	81 (22.4)	89 (22.1)	94 (25.3)	95 (27.6)
	Absolute Mean (SD) Change from baseline (mmHg)	-	20 (31.9)	27 (29.1)	32 (35.1)	34 (31.9)
	% improvement from Baseline	-	17.0	31.0	37.0	46.0
**4**	ABPI	0.47 (0.156)	0.61 (0.164)	0.67 (0.134)	0.70 (0.156)	0.73 (0.201)
	Absolute Mean (SD) increase from baseline	-	0.15 (0.226)	0.21 (0.200)	0.24 (0.209)	0.26 (0.211)
	% improvement from baseline	-	17.8	30.6	37.1	44.4
**5**	Total walking distance (m/h)	0.22 (0.254)	0.29 (0.218)	0.44 (0.319)	0.64 (0.555)	0.88 (1.104)
	Change (increase) from baseline. Mean (SD)	-	0.07 (0.132)	0.23 (0.283)	0.42 (0.458)	0.67 (1.100)
	% improvement from baseline	-	47.1	200.6	268.7	827.0

Abbreviations: ABPI, Ankle brachial pressure index

#### Phase IV study

Gupta et al. conducted an open label, multicenter phase IV post marketing surveillance study (CTRI/2018/02/011839), focusing on patients with CLTI resulting from Buerger’s disease. The study enrolled 50 adult patients diagnosed with CLTI caused by Buerger’s disease, as per Rutherford classification (Rutherford III-5 or III-6). These patients exhibited symptoms such as rest pain and/or ulcers in the affected limb and had either been ineligible for or had not responded to traditional revascularization treatment. They were administered REGENACIP^®^ at a dose of 2 million/Kg injected into the gastrocnemius muscle and around the ulcer site. The study participants were followed up for 12 months for efficacy and safety outcomes and 3y thereafter for safety outcome. The Clinical benefit of REGENACIP^®^ was similar to those reported in previous studies. Rest pain reduced gradually from a mean pain score of 7.8 at baseline to 1.4 at 12 months. Rest pain scores reduced significantly at rate of 0.45 unit per month over the period of 12 months (*P* < 0.0001) compared with baseline. At the 12-month, 73% of ulcers at baseline were completely healed, and REGENACIP^®^ therapy reduced the risk of developing new ulcers by 87% post treatment. Ankle systolic pressure and ABPI showed 73% and 71% increase respectively at 12 months post therapy. The efficacy outcomes with REGENACIP^®^ therapy in this study is summarized in Table 3; Fig. 5 [46].

**Table 3:** Summary of statistically significant change in efficacy outcomes at 12 months with REGENACIP^®^ therapy in phase IV study performed in CLTI patients due to Buerger’s disease (46). The change from baseline in each parameter is statistically significant at *P* ≤ 0.001

Sr No.	Efficacy parameter	Baseline	1 month	6 months	12 months
**1**	**Rest pain score (units)**	7.8 ± 0.2	4.4 ± 0.3	2.2 ± 0.3	1.4 ± 0.3
**2**	**Ankle systolic pressure (mm Hg)**	56.2 ± 2.2	79.3 ± 3.5	90.2 ± 4.0	92.3 ± 4.4
**3**	**ABPI**	0.44 ± 0.02	0.63 ± 0.03	0.74 ± 0.03	0.75 ± 0.04

Data expressed as mean ± SD

Abbreviations: ABPI, Ankle brachial pressure index

The Fontaine classification system assigns severity based entirely on clinical symptoms without other diagnostic tests, and Rutherford classification assigns severity based on performance on the 5-minute treadmill test at 2 mph on a 12% incline

Differentiation into replacement cell types and modulation of immune responses are the foundational mechanism for MSC rescue and/or repair function. The immunomodulatory modes include paracrine activity, cell–cell contact and interaction, mitochondrial transfer, and release of extracellular vesicles. MSC: Mesenchymal Stem Cell [34]

#### Safety and tolerability of REGENACIP therapy

Published data from cohort studies demonstrate a good safety profile with cell-based therapy in CLTI [46]. Safety of REGENACIP^®^ therapy has been studied in all phases of clinical trials for CLTI with underlying etiology of PAD or BD and the incidences of adverse events (AE) and serious adverse event (SAE) were remotely related or unrelated to REGENACIP^®^ therapy. None of the patients reported any clinically significant abnormalities in laboratory parameters or vital signs during the study period in each of the trials. The phase I/II study (*n* = 20 patients), conducted with the primary objective of assessment of safety showed similar safety profile between REGENACIP and placebo treatments (13 AE from 6 patients on REGENACIP^®^ Vs 45 AE from 8 patients on placebo, *p* = 0.6256; 6 SAE from REGENACIP^®^ arm Vs 8 SAE from placebo arm). Also, REGENACIP^®^ treatment did not adversely alter the immunological profiles in the treated patients as it did not elicit T cells proliferative response in vivo and comparable pro-inflammatory cytokines levels in both the cell and placebo arms at various time points [39]. The Phase II study in patients with CLTI due to BD (*n* = 90) demonstrated comparable safety profiles between the groups receiving 1 million cells/kg and 2 million cells/kg body weight dose and placebo group [44]. Similarly, in the phase III study involving CLTI patients with PAD (*n* = 24), REGENACIP^®^ therapy did not result in procedure-related complications, deaths or life-threatening SAE during the 12 months follow-up period [45]. In the phase IV study, patients with CLTI due to BD (*n* = 50) were followed up for 4 years to assess safety outcomes. Most of the AEs reported in the study were mild to moderate in nature with pyrexia and pain in extremities being the most commonly observed treatment emergent AE [46].

### Challenges with MSCs and future directions

Despite encouraging results from clinical trials and cohort studies, there are several barriers to its application of MSCs in CLI management. The specific molecular mechanistic aspects of therapeutic benefits of progenitor cells are not well understood. A meta-analysis study including 62 studies and 3546 patients’ data collected for 15 years demonstrated that MSC therapy was safe in different patients [37]. However there is a need for further research to assess the long-term safety and efficacy of this treatment. Additionally, the success of stem cell therapy relies on MSC effectively integrating and targeting diseased tissues to restore function and homeostasis. Even when dispensed directly to disease sites, MSC therapy is significantly hindered by issues with cell viability, retention, and limited or non-specific migration capacity [47, 48]. Heterogeneity in donor MSC can lead to persistent issues with immune compatibility between the recipient and the donor [49]. These challenges are exacerbated in diseased conditions, where factors such as tissue hypoxia, hyperglycemia, reduced blood flow, and widespread local inflammation can be inhospitable to MSC [50]. Thus, to achieve the maximum clinical efficacy, it is essential to optimize MSCs’ native functionality and delivery conditions while carefully considering the recipient tissue microenvironment where they are implanted [51]. Advances in regenerative medicine with angiogenesis and neovascularization from MSC-based therapy has been well demonstrated and is now emerging as a feasible therapeutic option in the context of disease management with failed revascularization or conditions where revascularization is not possible. However more multicenter clinical trials, real world evidence studies are required to further bolster confidence in these promising findings, establish with long term safety and efficacy with MSC therapy and elucidate aspects such as the best route of administration, the best MSC sources, the local environment affecting their performance and action, the special markers modulating the angiogenic response to propose the more optimized therapeutic strategies. Furthermore, future work should consider the ability of other cell-based therapies such as endothelial progenitor cells and mononuclear cells alone or in combination with MSCs for clinical revascularization strategies [33, 47–51].

## Conclusion

Peripheral Artery Disease is a significant disease and health care burden with prevalence varying from 5 to 25% and increases with age.  CLTI represents the most severe form and the end stage of PAD. Nearly 10% PAD patients suffer from CLTI but > 50% eventually become candidate to amputation and/or succumb to death due to cardiovascular causes. The traditional therapeutic approach of revascularization fails to alleviate the condition of CLTI in some patients who are then left with limited treatment options. Over the last decade, stem cell therapy has rapidly advanced and emerged as a therapeutic modality in conditions like CLTI where drugs have limited benefits and revascularization has failed. REGENACIP^®^ is a novel stem cell formulation derived from adult human bone marrow, cultured, pooled allogenic mesenchymal stromal cells which is one of the first stem cell-based biologics to be approved by CDSCO in patients with Atherosclerotic Peripheral Artery Disease patients or Buerger’s disease with established critical limb ischemia in Rutherford III-5 or III-6, not eligible for or have failed traditional revascularization treatment, with rest pain and / or ulcers in the affected limb (no option CLTI). The current literature as summarized in this review demonstrates that intramuscular administration of REGENACIP^®^ is safe, tolerable, and effective alternative to achieve therapeutic angiogenesis in patients with no option CLTI due to PAD or BD.

## Data Availability

Not applicable.

## References

[1] Eid MA, Mehta KS, Goodney PP. Epidemiology of peripheral artery disease. Semin Vasc Surg. 2021;34(1):38–46. 33757634 10.1053/j.semvascsurg.2021.02.005

[2] Correa-Posada MO, García-Velez JF, Hurtado-Mosquera OA, Sierra-Juárez MA, Hernández EF, Castillo CN, Barrera GE, Valderrama-Treviño AI. Thromboangiitis obliterans: a review. Int Surg J. 2024;11(6):1033–40.

[3] Barnes JA, Eid MA, Creager MA, Goodney PP. Epidemiology and risk of amputation in patients with diabetes mellitus and peripheral artery disease. Arterioscler Thromb Vasc Biol. 2020;40(8):1808–17. 32580632 10.1161/ATVBAHA.120.314595 PMC7377955

[4] Jones DW, Farber A. Review of the global vascular guidelines on the management of chronic limb-threatening ischemia. JAMA Surg. 2020;155(2):161–2. 31851292 10.1001/jamasurg.2019.4928

[5] Kinlay S. Management of critical limb ischemia. Circ Cardiovasc Interv. 2016;9(2):e001946. 26858079 10.1161/CIRCINTERVENTIONS.115.001946 PMC4827334

[6] Fowkes FGR, Aboyans V, Fowkes FJ, McDermott MM, Sampson UK, Criqui MH. Peripheral artery disease: epidemiology and global perspectives. Nat Rev Cardiol. 2017;14(3):156–70. 27853158 10.1038/nrcardio.2016.179

[7] Fowkes FGR, Rudan D, Rudan I, Aboyans V, Denenberg JO, McDermott MM, et al. Comparison of global estimates of prevalence and risk factors for peripheral artery disease in 2000 and 2010: a systematic review and analysis. Lancet. 2013;382(9901):1329–40. 23915883 10.1016/S0140-6736(13)61249-0

[8] Aday AW, Matsushita K. Epidemiology of peripheral artery disease and polyvascular disease. Circ Res. 2021;128(12):1818–32. 34110907 10.1161/CIRCRESAHA.121.318535 PMC8202714

[9] Horváth L, Németh N, Fehér G, Kívés Z, Endrei D, Boncz I. Epidemiology of peripheral artery disease: narrative review. Life. 2022;12(7):1041. 35888129 10.3390/life12071041 PMC9320565

[10] Lozano Navarro LV, Chen X, Giratá Viviescas LT, Ardila-Roa AK, Luna-Gonzalez ML, Sossa CL, et al. Mesenchymal stem cells for critical limb ischemia: their function, mechanism, and therapeutic potential. Stem Cell Res Ther. 2022;13(1):345. 35883198 10.1186/s13287-022-03043-3 PMC9327195

[11] Krishnan MN, Geevar Z, Mohanan PP, Venugopal K, Devika S. Prevalence of peripheral artery disease and risk factors in the elderly: a community based cross-sectional study from northern Kerala, India. Indian Heart J. 2018;70(6):808–15. 30580849 10.1016/j.ihj.2017.11.001 PMC6306488

[12] Eshcol J, Jebarani S, Anjana RM, Mohan V, Pradeepa R. Prevalence, incidence and progression of peripheral arterial disease in Asian Indian type 2 diabetic patients. J Diabetes Complications. 2014;28(5):627–31. 24930714 10.1016/j.jdiacomp.2014.04.013

[13] Narayana Health. What is peripheral arterial disease (PAD)? [Internet]. 2020. https://www.narayanahealth.org/blog/what-is-peripheral-arterial-disease-pad/

[14] Mary L, Yost. India – Peripheral Artery Disease and Critical Limb Ischemia 2018 [Internet]. THE SAGE GROUP; [cited 2023 Jul 22]. https://www.thesagegroup.us/reports/india-peripheral-artery-disease-and-critical-limb-ischemia-2018/

[15] Members WC, Gornik HL, Aronow HD, Goodney PP, Arya S, Brewster LP, Byrd L, Chandra V, Drachman DE, Eaves JM, Ehrman JK. 2024 ACC/AHA/AACVPR/APMA/ABC/SCAI/SVM/SVN/SVS/SIR/VESS guideline for the management of lower extremity peripheral artery disease: a report of the American College of Cardiology/American Heart Association Joint Committee on Clinical Practice guidelines. J Am Coll Cardiol. 2024;83(24):2497–604. 10.1016/j.jacc.2024.02.013 PMC12728805 38752899

[16] Akar AR, İnan MB, Baran Ç. Thromboangiitis Obliterans. Curr Treat Options Rheum. 2016;2:178–95.

[17] Qaja E, Muco E, Hashmi MF, Buerger Disease. [Updated 2023 Feb 19]. In: StatPearls [Internet]. Treasure Island (FL): StatPearls Publishing; 2024 Jan-. https://www.ncbi.nlm.nih.gov/books/NBK430858/28613608

[18] Puéchal X, Fiessinger J-N. Thromboangiitis obliterans or Buerger’s disease: challenges for the rheumatologist, Rheumatology, Volume 46, Issue 2, February 2007, Pages 192–199. 10.1093/rheumatology/kel388 17116654

[19] Dash C, Peyvandi B, Duan H, Richardson K, Ndon EU, Gabrick SS, Faz KA, Persing AA, Dardik J, Hsia AC. Stem cell therapy for thromboangiitis obliterans (Buerger’s disease). Processes. 2020;8(11):1408.

[20] Sasaki S, Sakuma M, Yasuda K. Current status of thromboangiitis obliterans (Buerger’s disease) in Japan. Int J Cardiol. 2000;75(Suppl 1):S175–81. 10980360 10.1016/s0167-5273(00)00190-x

[21] Golledge J. Update on the pathophysiology and medical treatment of peripheral artery disease. Nat Rev Cardiol. 2022;19(7):456–74. 34997200 10.1038/s41569-021-00663-9

[22] Patterson D, Belch JJ. Pathophysiology of critical limb ischemia. Vascular medicine. Elsevier; 2006. pp. 248–53.

[23] Halliday A, Bax JJ. The 2017 ESC guidelines on the diagnosis and treatment of peripheral arterial diseases, in collaboration with the European Society for Vascular Surgery (ESVS). Eur J Vasc Endovasc Surg. 2018;55(3):301–2. 29579461 10.1016/j.ejvs.2018.03.004

[24] Hardman RL, Jazaeri O, Yi J, Smith M, Gupta R. Overview of classification systems in peripheral artery disease. Semin Intervent Radiol. 2014;31(4):378–88. 25435665 10.1055/s-0034-1393976 PMC4232437

[25] Bradbury AW, Adam DJ, Bell J, Forbes JF, Fowkes FG, Gillespie I, Ruckley CV, Raab GM, BASIL trial Participants. Bypass versus angioplasty in severe ischaemia of the Leg (BASIL) trial: an intention-to-treat analysis of amputation-free and overall survival in patients randomized to a bypass surgery-first or a balloon angioplasty-first revascularization strategy. J Vasc Surg. 2010;51(5 Suppl):S5–17. 10.1016/j.jvs.2010.01.073. Erratum in: J Vasc Surg. 2010;52(6):1751. Bhattachary, V [corrected to Bhattacharya, V]. PMID: 20435258. 10.1016/j.jvs.2010.01.073 20435258

[26] Armstrong EJ, Alam S, Henao S, Lee AC, DeRubertis BG, Montero-Baker M, et al. Multidisciplinary care for critical limb ischemia: current gaps and opportunities for improvement. J Endovasc Ther. 2019;26(2):199–212. 30706755 10.1177/1526602819826593

[27] Mustapha JA, Finton SM, Diaz-Sandoval LJ, Saab FA, Miller LE. Percutaneous transluminal angioplasty in patients with infrapopliteal arterial disease: systematic review and meta-analysis. Circ Cardiovasc Interv. 2016;9(5):e003468. 27162214 10.1161/CIRCINTERVENTIONS.115.003468

[28] Stem Cell Basics [internet]. https://stemcells.nih.gov/info/basics/stc-basics. Accessed on 19th July 2024.

[29] Sullivan DC, Bicknell R. New molecular pathways in angiogenesis. Br J Cancer. 2003;89(2):228–31. 12865906 10.1038/sj.bjc.6601107 PMC2394258

[30] Müller L, Tunger A, Wobus M, Von Bonin M, Towers R, Bornhäuser M, et al. Immunomodulatory properties of mesenchymal stromal cells: an update. Front Cell Dev Biol. 2021;9:637725. 33634139 10.3389/fcell.2021.637725 PMC7900158

[31] Shirbaghaee Z, Hassani M, Heidari Keshel S, Soleimani M. Emerging roles of mesenchymal stem cell therapy in patients with critical limb ischemia. Stem Cell Res Ther. 2022;13(1):462. 36068595 10.1186/s13287-022-03148-9 PMC9449296

[32] Maacha S, Sidahmed H, Jacob S, Gentilcore G, Calzone R, Grivel JC, et al. Paracrine mechanisms of mesenchymal stromal cells in Angiogenesis. Stem Cells Int. 2020;2020:1–12. 10.1155/2020/4356359 PMC7085399 32215017

[33] Lozano Navarro LV, Chen X, Giratá Viviescas LT, Ardila-Roa AK, Luna-Gonzalez ML, Sossa CL, Arango-Rodríguez ML. Mesenchymal stem cells for critical limb ischemia: their function, mechanism, and therapeutic potential. Stem Cell Res Ther. 2022;13(1):345. 35883198 10.1186/s13287-022-03043-3 PMC9327195

[34] Česnik AB, Švajger U. The issue of heterogeneity of MSC-based advanced therapy medicinal products-a review. Front Cell Dev Biol. 2024;12:1400347. 39129786 10.3389/fcell.2024.1400347 PMC11310176

[35] Chouaib B, Haack-Sørensen M, Chaubron F, Cuisinier F, Collart-Dutilleul PY. Towards the standardization of Mesenchymal Stem Cell Secretome-Derived Product Manufacturing for tissue regeneration. Int J Mol Sci. 2023;24(16):12594. 37628774 10.3390/ijms241612594 PMC10454619

[36] Volarevic V, Markovic BS, Gazdic M, Volarevic A, Jovicic N, Arsenijevic N, Armstrong L, Djonov V, Lako M, Stojkovic M. Ethical and Safety issues of Stem Cell-based therapy. Int J Med Sci. 2018;15(1):36–45. 29333086 10.7150/ijms.21666 PMC5765738

[37] Wang Y, Yi H, Song Y. The safety of MSC therapy over the past 15 years: a meta-analysis. Stem Cell Res Ther. 2021;12:1–5. 34663461 10.1186/s13287-021-02609-x PMC8522073

[38] Zocchi ML, Vindigni V, Pagani A, Pirro O, Conti G, Sbarbati A, Bassetto F. Regulatory, ethical, and technical considerations on regenerative technologies and adipose-derived mesenchymal stem cells. Eur J Plast Surg. 2019;42:531–48.

[39] Lahiry S, Choudhury S, Sinha R, Chatterjee S. The national guidelines for stem cell research (2017): what academicians need to know? Perspect Clin Res. 2019;10(4):148–54. 31649863 10.4103/picr.PICR_23_18 PMC6801994

[40] https://dbtindia.gov.in/sites/default/files/National_Guidelines_StemCellResearch-2017.pdf Assessed on 5th September 2024.

[41] Assen LS, Jongsma KR, Isasi R, Tryfonidou MA, Bredenoord AL. Recognizing the ethical implications of stem cell research: a call for broadening the scope. Stem cell Rep. 2021;16(7):1656–61. 10.1016/j.stemcr.2021.05.021 PMC8282461 34214488

[42] Prescribing information REGENACIP ^®^Cipla Pvt Ltd. year 2021. https://www.ciplamed.com/product-index/REGENACIP®-150m-injection-for-cli-pad; Accessed on 27 Nov 2023.

[43] Gupta PK, Chullikana A, Parakh R, Desai S, Das A, Gottipamula S, et al. A double-blind randomized placebo-controlled phase I/II study assessing the safety and efficacy of allogeneic bone marrow derived mesenchymal stem cell in critical limb ischemia. J Transl Med. 2013;11:1–11. 23758736 10.1186/1479-5876-11-143 PMC3688296

[44] Gupta PK, Krishna M, Chullikana A, Desai S, Murugesan R, Dutta S, et al. Administration of adult human bone Marrow-Derived, cultured, pooled, allogeneic mesenchymal stromal cells in critical limb ischemia due to Buerger’s Disease: phase II study report suggests clinical efficacy. Stem Cells Transl Med. 2017;6(3):689–99. 28297569 10.5966/sctm.2016-0237 PMC5442769

[45] Gupta PK, Shivashankar P, Rajkumar M, Mahapatra SS, Desai SC, Dhar A, et al. Label extension, single-arm, phase III study shows efficacy and safety of stempeucel^®^ in patients with critical limb ischemia due to atherosclerotic peripheral arterial disease. Stem Cell Res Ther. 2023;14(1):60. 37005673 10.1186/s13287-023-03292-w PMC10068167

[46] Gupta PK, Dutta S, Kala S, Nekkanti M, Desai SC, Mahapatra SS, et al. Phase IV Postmarketing Surveillance Study shows continued efficacy and safety of Stempeucel in patients with critical limb ischemia due to Buerger’s Disease. Stem Cells Transl Med. 2021;10(12):1602–13. 34519179 10.1002/sctm.21-0197 PMC8641082

[47] Kwiatkowski T, Zbierska-Rubinkiewicz K, Krzywoń J, Szkółka Ł, Kuczmik W, Majka M, Maga P, Drelicharz Ł, Musiałek P, Trystuła M. Cellular therapies in no-option critical limb ischemia: present status and future directions. Adv Interventional Cardiology/Postępy w Kardiologii Interwencyjnej. 2022;18(4):340–9. 10.5114/aic.2022.120962 PMC10031679 36967860

[48] Liu ZJ, Daftarian P, Kovalski L, Wang B, Tian R, Castilla DM, et al. Directing and potentiating stem cell-mediated angiogenesis and tissue repair by cell surface E-selectin coating. PLoS ONE. 2016;11:e0154053. 27104647 10.1371/journal.pone.0154053 PMC4841581

[49] Zhou T, Yuan Z, Weng J, Pei D, du X, He C, et al. Challenges and advances in clinical applications of mesenchymal stromal cells. J Hematol Oncol. 2021;14:24. 33579329 10.1186/s13045-021-01037-x PMC7880217

[50] Huerta CT, Voza FA, Ortiz YY, Liu ZJ, Velazquez OC. Mesenchymal stem cell-based therapy for non-healing wounds due to chronic limb-threatening ischemia: a review of preclinical and clinical studies. Front Cardiovasc Med. 2023;10:1113982. 36818343 10.3389/fcvm.2023.1113982 PMC9930203

[51] Jeyaraman M, Nagarajan S, Maffulli N, Packkyarathinam RP, Jeyaraman N, Arulkumar N, Khanna M, Yadav S, Gupta A. Stem cell therapy in critical limb ischemia. Cureus. 2023;15(7). 10.7759/cureus.41772 PMC10416751 37575721

